# Obstacle traversal and route choice in flying honeybees: Evidence for individual handedness

**DOI:** 10.1371/journal.pone.0184343

**Published:** 2017-11-02

**Authors:** Marielle Ong, Michael Bulmer, Julia Groening, Mandyam V. Srinivasan

**Affiliations:** 1 Queensland Brain Institute, University of Queensland, Brisbane, Queensland, Australia; 2 School of Mathematics and Physics, University of Queensland, Brisbane, Queensland, Australia; 3 School of Information Technology and Electrical Engineering, University of Queensland, Brisbane, Queensland, Australia; Colorado State University, UNITED STATES

## Abstract

Flying insects constantly face the challenge of choosing efficient, safe and collision-free routes while navigating through dense foliage. We examined the route-choice behavior of foraging honeybees when they encountered a barrier which could be traversed by flying through one of two apertures, positioned side by side. When the bees’ choice behavior was averaged over the entire tested population, the two apertures were chosen with equal frequency when they were equally wide. When the apertures were of different width, the bees, on average, showed a preference for the wider aperture, which increased sharply with the difference between the aperture widths. Thus, bees are able to discriminate the widths of oncoming gaps and choose the passage which is presumably safer and quicker to transit. Examination of the behavior of individual bees revealed that, when the two apertures were equally wide, ca. 55% of the bees displayed no side bias in their choices. However, the remaining 45% showed varying degrees of bias, with one half of them preferring the left-hand aperture, and the other half the right-hand aperture. The existence of distinct individual biases was confirmed by measuring the times required by biased bees to transit various aperture configurations: The transit time was longer if a bee’s intrinsic bias forced it to engage with the narrower aperture. Our results show that, at the population level, bees do not exhibit ‘handedness’ in choosing routes; however, individual bees display an idiosyncratic bias that can range from a strong left bias, through zero bias, to a strong right bias. In honeybees, previous studies of olfactory and visual learning have demonstrated clear biases at the population level. To our knowledge, our study is the first to uncover the existence of individually distinct biases in honeybees. We also show how a distribution of biases among individual honeybees can be advantageous in facilitating rapid transit of a group of bees through a cluttered environment, without any centralized decision-making or control.

## Introduction

Foraging insects, such as honeybees, not only need to learn routes to bountiful food sources and fly to them repeatedly–they also need to be able to cope effectively with obstacles that are encountered en route, and choose between alternative passages to the destination [1, 2]. When faced with a choice between a wider passage and a narrower one, which passage will a honeybee choose? Previous studies have found that bees fly slower in narrower passages [3, 4], which might suggest that choosing the wider passage–apart from reducing the risk of injury (e.g. wing damage: [5])—would expedite the journey to the destination. Do bees discriminate between apertures of different widths? And, if so, how acute is their discrimination?

Another question this study explores, is whether bees choose randomly between two equally wide apertures presented side by side, or whether they exhibit a preference for one of them. If they do display a side-bias, does this bias prevail in the majority of the population (as in right-handedness in humans, for example) or does it vary with the individual, with some bees preferring the left-hand aperture and others the right? A substantial body of literature suggests that in many animal species, especially social ones, lateralization of various aspects of behavior is manifest at the population level, with most individuals displaying the same bias polarity (e.g. [6–10]). However, recent evidence is beginning to suggest that this is not always the case, even in social species: In many instances lateralization can vary with the individual, and also with the task at hand, for example ladybirds [11] and Drosophila [12] show turning biases that vary across individuals. A recent study has revealed that budgerigars, a highly social species of bird, exhibit a left bias at the population level when choosing between two landing perches, but individually varying side preferences when they approach and land on a long perch [13]. Moreover, it has been shown that budgerigars display individually distinct biases when choosing between two apertures of equal width–with some birds consistently favouring the left-hand aperture, others consistently the right, and yet others showing no preference [14]. It has been suggested that this variation of bias across individuals could expedite the passage of a flock of birds through a densely cluttered environment [14]. It is of interest, therefore, to consider similar scenarios with bees, which may need to choose between alternative routes when visiting or returning from a popular food source in large numbers, or when moving in swarms through dense vegetation. This question is of particular interest because there is some evidence that bees optimize their foraging routes [15] and maximize energetic efficiency [16].

Here we examine the behavior of foraging honeybees when they are offered a choice to fly through one of two apertures, positioned side by side, on their way to a food source. The side preferences of individual bees are measured as the relative widths of the two apertures are systematically varied from (narrow left, wide right), through (equal widths) to (wide left, narrow right). The data are analyzed using quantitative statistical models to examine whether the bees (a) display no left or right bias, and always prefer the wider aperture; or (b) display a population bias, in which the majority of bees tends to prefer one side (left or right) even when the apertures are of equal width; or (c) display individually varying biases, with some individuals preferring the left aperture, others preferring the right aperture, and yet others exhibiting no preference. We further investigate the effect of aperture configuration and side bias on the transit time, i.e. the time required by a bee to choose one of the apertures and fly through it, as this is an ecologically relevant feature of the bees’ flight performance.

In honeybees, lateralization of behavior and brain function has been investigated primarily with respect to the olfactory sense [17–19]. To our knowledge, this is the first study to investigate lateralization of route choices in foraging honeybees, with the aim of gaining a better understanding of the factors that influence route choices, and, more generally, to further increase our knowledge about the significance of ‘handedness’ in invertebrates. We find clear evidence for the existence of individually varying lateralization of behavior in honeybees. We suggest that a distribution of biases across individual honeybees can facilitate rapid transit of a group of bees through a cluttered environment.

## Materials and methods

### Subjects and study site

The experiments were performed at the Queensland Brain Institute of the University of Queensland in Brisbane, Australia. Honeybee foragers (*Apis mellifera*) were recruited from a hive located inside the institute’s climate controlled Bee Flight Facility (200 square metres, temperature: 24°C, humidity: 60%). The hive was connected to the outdoors via a Perspex tube, allowing bees to fly and forage wherever and whenever they pleased.

121 foragers were marked individually with acrylic (Derivan Matisse, Australia) and enamel paint (Tamiya, Japan), by placing colored dots on the thorax and/or the abdomen. Of these, 102 bees were used to investigate and analyze route choice behavior. (The remaining 19 individuals were not analyzed because of their uncertain identity, caused by fading of their markings). The transit times (described below) were recorded for 45 individually marked bees.

### Experimental setup

**Fig 1:**
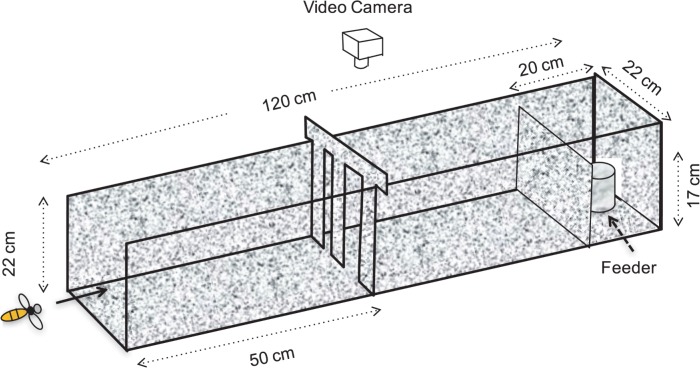
Experimental setup: Schematic view. Schematic view of the experimental setup (not to scale), showing the tunnel, the partition presenting the two apertures, the feeder, the barrier occluding the feeder, and the position of the overhead video camera.

Bees were trained to enter the Bee Flight Facility and fly through a tunnel (120 cm long, 22 cm wide and 22 cm high), made from corflute and incorporating a transparent Perspex ceiling (Fig 1). A feeder containing sucrose solution was placed at the end of the tunnel as a reward for the visiting foragers. The feeder was hidden behind a 17 cm high barrier to prevent the bees’ choice behavior from being affected by the feeder’s position. The walls, barrier and floors of the tunnel were covered with a red-white checkerboard texture (check size 2.2 cm x 2.2 cm) to provide visual guidance to the bees. The glass roof and windows of the facility were modified to provide diffuse illumination with no direct view of the sun, in order to not affect the bees’ flight behavior and aperture choice.

During the experiment, a transverse wall, carrying two vertically oriented apertures, was temporarily placed halfway along the tunnel (Fig 1). A visiting bee had to choose to fly through one of these apertures before proceeding to the feeder. After feeding, the bee re-encountered the two apertures, and again had to choose to fly through one of them before exiting the tunnel and returning to the hive. The bees’ route choices—during Entry as well as Exit—were recorded for various aperture configurations, as described below.

The width of each aperture was varied from a minimum of 2 cm to a maximum of 8 cm, in steps of 1 cm, whilst keeping the sum of the two widths constant at 10 cm. Four inserts were created, to produce (left, right) aperture configurations of (2 cm, 8 cm), (3 cm, 7 cm), (4 cm, 6 cm), and (5 cm, 5 cm). The remaining set of aperture configurations (with the wider opening on the left) were created by a left-right flip of the three asymmetrical inserts. The inserts were presented to the bees in a random sequence determined by a random number generator in the statistical computing software R, and were changed every five minutes. It should be noted that an insert, which presented for example a 4 cm aperture on the left and a 6 cm aperture on the right to an entering bee, would present the reverse configuration to an exiting bee.

### Scoring and analysis of route choice behavior

A bee’s choice was recorded only if there were no other bees present within a space 8.8 cm (4 check widths) on either side of the aperture insert. This ensured that the bee’s decision was not affected by the presence of other bees in the vicinity. The dimension of this space was chosen based on the bees’ body size, density of bee traffic and feasibility of visually monitoring the area.

Only individuals that had completed at least 10 Entry and 10 Exit flights were included in the data set. If a bee chose to fly through the right-hand aperture n times out of a total of N flights for a particular aperture configuration, then the probability of choosing the right-hand aperture for that particular bee and aperture configuration is n/N. This allowed the choices of bees to be examined on an individual basis. Each bee’s choices were evaluated separately for the Entry condition and the Exit condition, to investigate possible differences in choice behavior between their outbound and homebound journeys. The bees’ choices during Entry and Exit were scored as ‘left’ or ‘right’ with respect to their own reference frame (flight direction), and not with respect to the external geometry of the tunnel.

**Fig 2:**
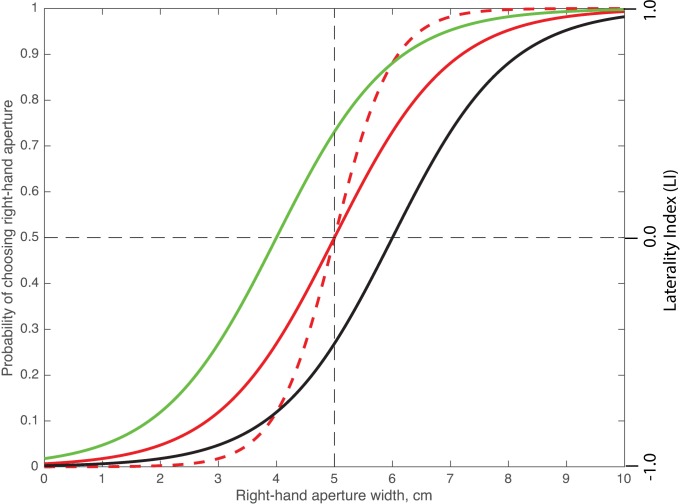
Probability of choosing the right-hand aperture as a function of its width. The figure shows example logistic functions for zero bias, aperture sensitivity = 1.0 ($\beta_{A}=1.0,\frac{\beta_{0}}{\beta_{A}}=-5.0$; solid red curve); zero bias, aperture sensitivity = 2.0 ($\beta_{A}=2.0,\frac{\beta_{0}}{\beta_{A}}=-5.0$; dashed red curve); right bias, aperture sensitivity = 1.0 ($\beta_{A}=1.0,\frac{\beta_{0}}{\beta_{A}}=-4.0$; solid green curve); left bias, aperture sensitivity = 1.0 ($\beta_{A}=1.0,\frac{\beta_{0}}{\beta_{A}}=-6.0$; solid black curve). The ordinate on the right in this figure and in Figs 3 and 4 shows the Laterality Index LI, as defined in ‘Materials and methods’.

**Fig 3:**
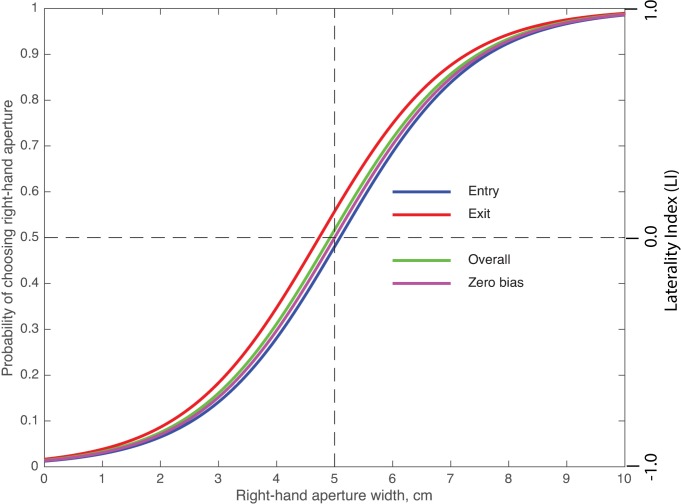
Variation of the bees’ preference for the right-hand aperture as a function of its width. Data are shown for the Entry condition (blue curve), the Exit condition (red curve), and both conditions combined (Overall: green curve). The curves represent fits of sigmoidal functions to the data, as described in ‘Materials and methods’. The purple curve depicts a theoretical zero-bias response function.

**Fig 4:**
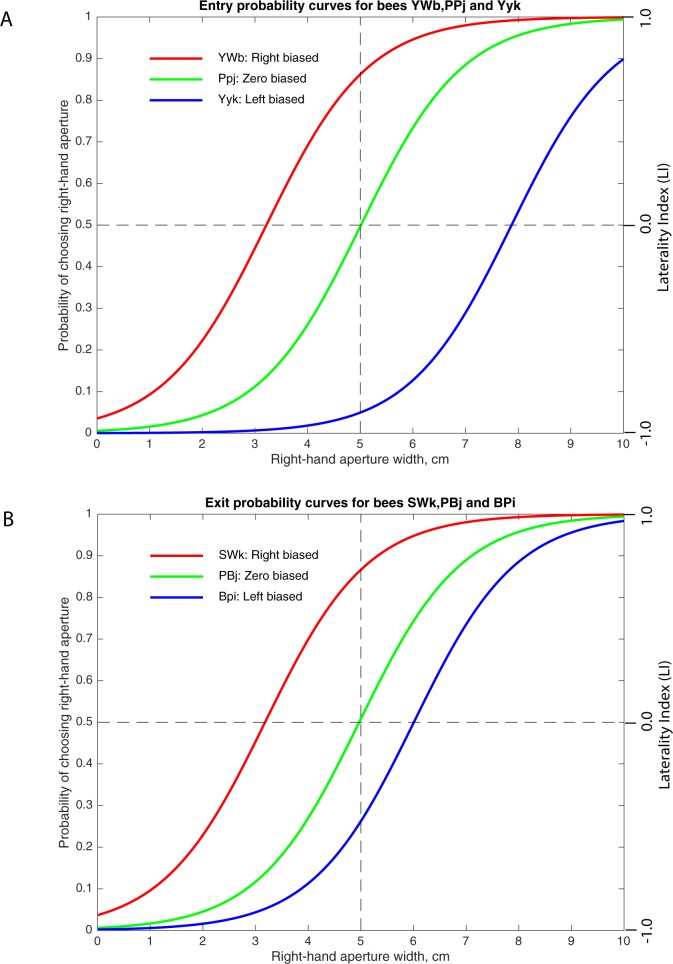
Logistic functions for the right aperture preferences of three individual bees. **A.** Logistic functions fitted for bees with a right bias (YWb), zero bias (PPj) and left bias (Yyk), for the Entry condition. **B.** Logistic functions fitted for bees with a right bias (SWk), zero bias (PBj), and left bias (BPi), for the Exit condition.

The plots in Figs 2–4 show the bees’ choices expressed as probabilities, as well as in terms of the ‘Laterality Index’ (LI). This is another commonly used measure of bias, which is computed as LI = (R-L)/(R+L), where R and L are the numbers of choices made by the bee for the right and left-hand apertures, respectively. LI assumes a value of 1.0 when the right-hand aperture attracts all of the choices, a value of -1.0 when the left-hand aperture attracts all of the choices, and a value of 0.0 when both apertures are equally attractive.

The data from individuals were pooled and analyzed using a statistical program in R that implemented polychotomous multiple logistic regression analysis to evaluate four different mathematical models, as described in S1 Appendix.

The logistic function described by Model 1 (the simplest model) is defined by

$$p=\frac{1}{\left[1+e^{{-\beta }_{A}(x+\frac{\beta_{0}}{\beta_{A}})}\right]}$$ (1)

where p denotes the probability of choosing the right-hand aperture, x denotes the width of the right-hand aperture (cm), β_A_ characterises the sensitivity to changes in aperture width (units of probability/cm), and the parameter $\frac{\beta_{0}}{\beta_{A}}$ (cm) is a measure of any bias in the choices (e.g. the right-hand aperture is preferred over the left-hand aperture even when the two apertures are equally wide). The parameter $-\frac{\beta_{0}}{\beta_{A}}$ represents the width of the right-hand aperture for which the two apertures are chosen with equal probabilities (of 0.5). The logistic function is illustrated in Fig 2 for a few different parameter values. If $-\frac{\beta_{0}}{\beta_{A}}$ = 5.0, the choices are unbiased; if $-\frac{\beta_{0}}{\beta_{A}}$ < 5.0 the choices are right-biased, and if $-\frac{\beta_{0}}{\beta_{A}}$ > 5.0 the choices are left-biased.

**Fig 5:**
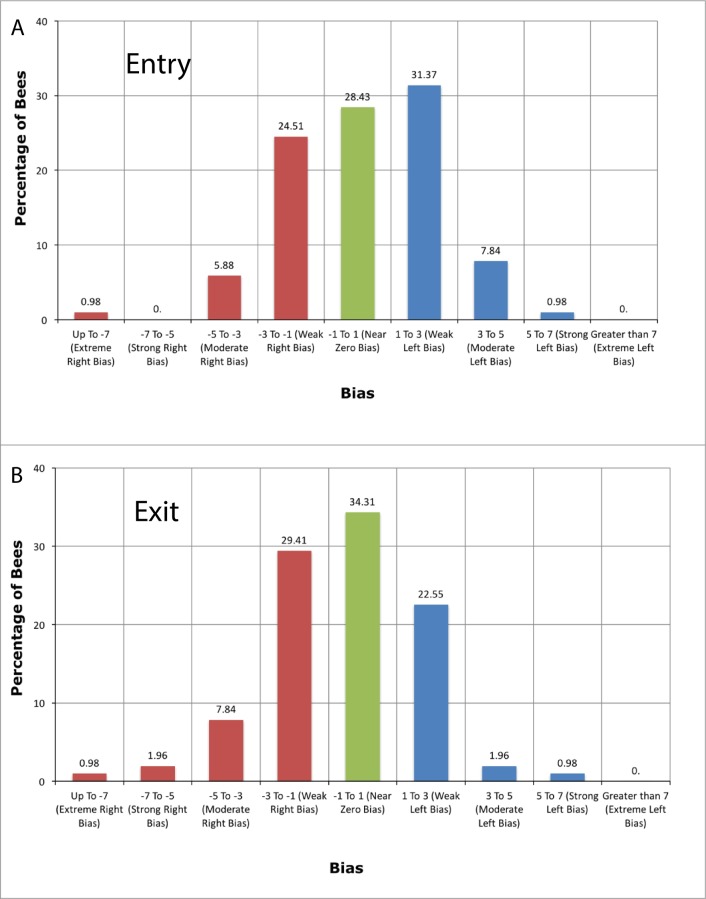
Histogram of biases of all bees. Biases of all of the 102 bees, tested for **(A)** the Entry condition and **(B)** the Exit condition. The polarity and magnitude of the bias of each bee is quantified by the parameter $\left[-\frac{\beta_{0}}{\beta_{A}}-5.0\right]$ (see ‘Materials and methods‘).

In Table 1 and Fig 5 we use the parameter $\left[-\frac{\beta_{0}}{\beta_{A}}-5.0\right]$ to quantify the polarity and magnitude of the bias. This parameter is positive if the choices are left-biased, zero if they are unbiased, and negative if they are right-biased. The magnitude of the parameter (which can vary from 0 to 7) represents the strength of the bias.

**Table 1:** Parameters defining the sigmoidal functions fitted to the data.

Condition	*β*_0_	*β*_*A*_	Bias = $\left(-\frac{\beta_{0}}{\beta_{A}}-5.0\right)$
Entry condition	-4.3736***	0.8590***	+0.0915 cm (Left bias, n.s.)
Exit condition	-4.0999***	0.8661***	-0.2663 cm (Right bias, n.s.)
Overall	-4.2330***	0.8602***	-0.0781 cm (Right bias, n.s.)
Theoretical zero bias	-4.3010	0.8602	0.0000 cm (Zero bias)

***β***_**0**_: Intercept parameter; ***β***_***A***_: Aperture parameter

***: P < 2E-16

### Estimation of confidence intervals of choice probabilities

**Fig 6:**
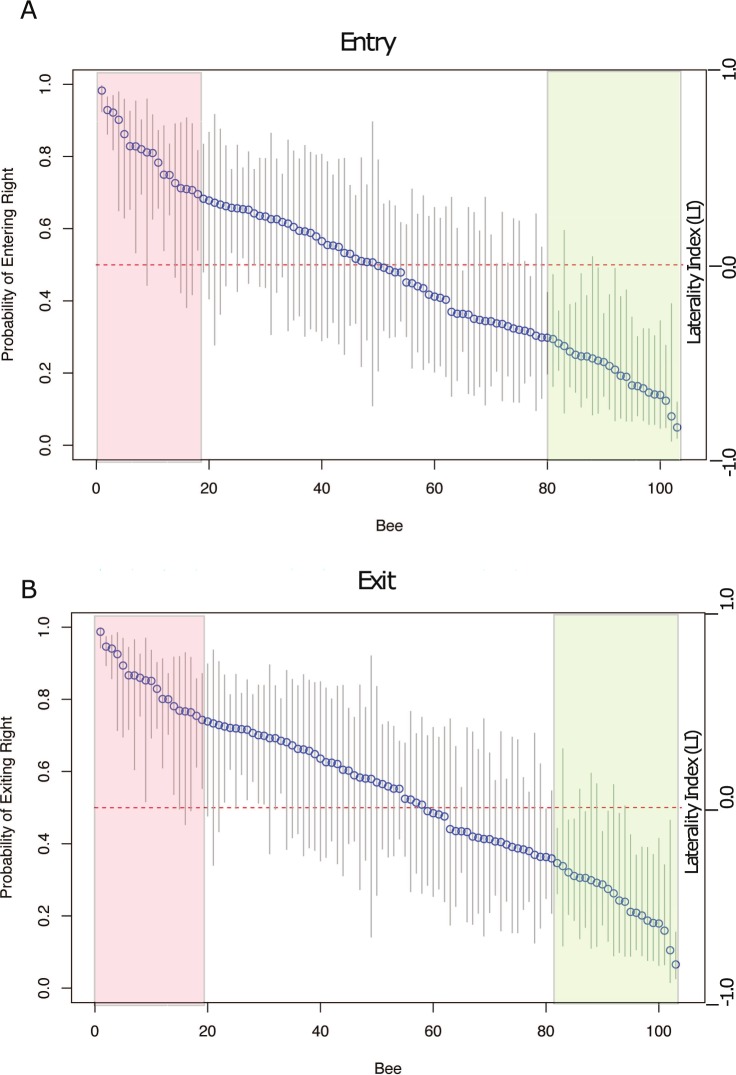
**Equal apertures: Probability of choosing the right-hand aperture for individual bees for (A) the Entry condition, and (B) the Exit condition.** Blue circles represent mean values, and grey vertical lines show 95% confidence intervals. The numbers on the horizontal axis represent individual bees, ranked according to their preference for the right-hand aperture. The horizontal dashed red line represents a random choice probability of 0.5. The red and green areas highlight bees with significant right and left biases, respectively.

In addition to analyzing parameter values, the multiple logistic regression model was used to calculate estimated choice probabilities under specific conditions, including for individual bees. Using the standard errors for these estimates, 95% confidence intervals were calculated from the model and then transformed with the logistic function to give confidence intervals for the choice probabilities. These confidence bands are displayed in Fig 6.

### Transit times: Filming and analysis

To analyze the transit times for different aperture configurations, the flights of 45 individually marked bees were recorded with a Samsung HD digital camcorder (VP-HMX20C). These bees represent a random subset of the tested population, as time constraints did not permit recording and analysis of transit times for all 102 individuals. The camera faced downwards above the aperture barrier and covered a 70.4 cm long section of the tunnel (Fig 1). Flights were recorded in 10 minute segments at 25 fps and analyzed using Quick Time Player and ImageJ. The transit zone was defined as the area spanning the entire width and height of the tunnel over a distance of 8.8 cm (4 check widths, see above) immediately before the aperture barrier. The transit time was measured as the time (in frames) that elapsed between the bee’s entry into this section, and its exit via the chosen aperture.

## Results

### Discrimination of aperture width

The bees’ aperture preferences in relation to their relative widths were analyzed separately for individuals that were entering the tunnel on the way to the food source (Entry condition; blue curve, Fig 3), and for bees that were leaving the tunnel after feeding and encountering the aperture from the opposite direction (Exit condition; red curve, Fig 3). These two data-sets were pooled to obtain the overall choice behavior of the bees, irrespective of Entry or Exit (Overall condition; green curve, Fig 3). These response curves represent the choice probability for the right-hand aperture and were obtained by fitting sigmoidal functions to the data, as described in Eq (1) in ‘Materials and methods’. Also shown for comparison is the theoretical curve for a zero bias function (purple curve, Fig 3) with the same slope as the population curve.

The parameters defining the four curves are given in Table 1, which also shows the bias computed for each condition, as described in ‘Materials and methods’. The bias represents the difference in aperture widths (right-hand aperture minus left-hand aperture) at which the two apertures are used equally often and none of them is preferred. For example, in the Entry condition, this would occur in theory when the right aperture is 5.09 cm and the left aperture is 4.91 cm, signifying a marginal left bias.

Fits are shown for the Entry condition, the Exit condition, the Overall condition, and a theoretical zero bias function with the same value of β_A_ (slope) as the population function. (***) denotes a significance level of P < 2E-16 for the probability that the measured parameter is non-zero.

How do the bees’ choices of the two apertures vary if their relative widths are changed? Let us first consider the response curve for the overall population (green). It is evident that, when the population is regarded as a whole, the bees choose the left and right apertures approximately equally often when they are of equal width. In other words, the bees do not display a noticeable bias, or preference for one side over the other. However, they do prefer the right-hand aperture when it is wider than the left-hand aperture, and vice versa. The fitted value of the parameter *β*_*A*_ of the logistic function has units of probability/cm and describes its steepness (i.e. the ability to discriminate between apertures of unequal width). *β*_*A*_ has a value of 0.8602/cm, which implies that the bees possess a good capacity to discriminate aperture widths–when one aperture (6.67 cm) is twice as wide as the other aperture (3.33 cm), the wider aperture is chosen about 80% of the time (Fig 3). The response curve for the overall population resembles very closely the theoretically expected curve for a population of bees with zero average bias (purple) but the population curve (green) is shifted by 0.08 cm to the left. However, this small displacement does not represent a significant right bias of the overall population—the mean value of the biases estimated for all of the bees is not significantly different from zero (P = 0.597, t-test). Statistical comparison of the fit of the theoretical zero-bias curve to the data (Table 2) also shows that there is no significant difference between the measured choice probabilities and the zero-bias curve at any of the aperture widths. A Z-score test reveals that, when the apertures are equally wide, the probability of choice of the right-hand aperture is not significantly different from 0.5 (P = 0.288), again confirming no significant bias.

**Table 2:** Statistical analysis of the overall population response curve (green) of Fig 1.

Right-hand aperture width	2.0 cm	3.0 cm	4.0 cm	5.0 cm	6.0 cm	7.0 cm	8.0 cm
Measured choice probabilities	0.075	0.161	0.312	0.517	0.717	0.857	0.934
Zero bias function choice probabilities	0.070	0.152	0.297	0.5	0.703	0.848	0.930
Standard error (S.E.)	0.011	0.014	0.019	0.021	0.018	0.014	0.010
Z-score (proportion test)	0.430	0.636	0.738	0.800	0.759	0.614	0.423
p-values	0.166	0.239	0.270	0.288	0.276	0.229	0.163

Comparison of the measured choice probabilities with the theoretical values expected from a zero-bias curve, using a proportion test.

Table 2 shows a comparison of the measured choice probabilities with the theoretical values expected from a zero-bias curve (purple, Fig 3), using a proportion test. There is no significant difference between the two curves at any of the relative aperture widths.

Considering the Entry and Exit data separately, we find that the response curve for the Entry condition is shifted by 0.09 cm to the right of the zero-bias curve. However, this displacement again does not represent a significant left bias (P = 0.567, t-test). The response curve for the Exit condition is shifted to the left by 0.27 cm. Again, this shift does not represent a significant right bias (P = 0.102, t-test). There is, however, a significant difference in bias between the Entry and the Exit conditions (P < 0.00001, t-test). But, while this difference is statistically significant, its magnitude is small (0.36 cm).

We may summarize the above findings as follows: When the two apertures are of equal width, they are chosen with the same frequency, on average. When one aperture is wider, the bees show a tendency to prefer it. The preference for the wider opening increases as the difference between the aperture widths increases, and demonstrates that bees possess a good capacity to discriminate gap widths–the wider aperture is chosen 80% of the time when it is twice as wide as the other aperture. This is true for the Entry and Exit condition, as well as the overall population.

For equally wide apertures the tested population of 102 bees, considered as a whole, is unbiased and does not exhibit any significant left or right preference. The reasons for this may be (a) none of the bees is biased; (b) half of the bees are left-biased, while the other half are right-biased; or (c) some bees are left-biased, others are right-biased, and a third group is unbiased. We investigated this question by looking at the choice behavior of individual foragers.

### Biases of individual bees–Method 1

The biases of individual bees were determined using two different methods. In Method 1, we calculated the biases of all 102 individuals for the Entry condition and the Exit condition by fitting each bee’s data (choice behavior for each of the aperture configurations) to a logistic function as described in ‘Materials and methods’ and evaluating the bias as $\left(-\frac{\beta_{0}}{\beta_{A}}-5.0\right)$. This parameter is positive if the choices are left-biased, and negative if the choices are right-biased. The magnitude of the parameter (which can vary from 0 to 7) represents the strength of the bias.

This analysis revealed that the bias can vary from one individual to the next, in direction as well as strength. As an example, the response curves for three bees for the Entry condition are shown in Fig 4A. Bee PPj exhibits a near-zero bias, whereas YWb displays a clear right bias and YYk has a clear left bias. The two latter bees displayed the same bias polarity during Entry as well as Exit. Their measured bias parameters were: YWb: -1.78 Entry, -2.07 Exit; YYk: 2.87 Entry, 2.59 Exit. Similarly, Fig 4B shows the response curves for three individuals for the Exit condition, with bee PBj displaying a near-zero bias, whereas SWk has a clear right bias and Bpi exhibits a clear left bias.

The majority of individuals (93 of 102 bees) displayed an individually consistent bias across the Entry and Exit conditions. This demonstrates that the variation in the biases observed across different individuals was real, and was not caused by artefacts such as uneven illumination or asymmetry in the geometry of the apparatus.

The small number of individuals whose bias changed between Entry and Exit (9 out of 102 bees) were all left biased during Entry, and right biased during Exit. These bees, however, were found to have biases close to zero, ranging from 0.02 cm to 0.47 cm during Entry and -0.06 cm to -0.55cm during Exit.

The spread of biases across the entire population is shown in Fig 5A for the Entry condition, and in Fig 5B for the Exit condition. The polarity and magnitude of the bias are specified by the parameter $\left[-\frac{\beta_{0}}{\beta_{A}}-5.0\right]$, as described in ‘Materials and methods’. This parameter is positive if the choices are left-biased, zero if they are unbiased, and negative if they are right-biased. The magnitude of the parameter (which can vary from 0 to 7) represents the strength of the bias.

For both the Entry and the Exit conditions, approximately 30% of the bees display a bias that is close to zero, while the remaining 70% show weak to extreme biases to the left or the right.

### Biases of individual bees–Method 2

Next, we examined the biases displayed by individual bees when the apertures were of equal width. Since each individual made at least 10 choices in the equal aperture condition, it was possible to obtain a choice probability for the right-hand aperture for each bee, and a confidence band for this probability, as described in ‘Materials and methods’.

The results for the Entry condition are displayed in Fig 6A, where the horizontal axis ranks individual bees according to their choice probability for the right-hand aperture, and the vertical axis shows the individual choice probabilities and their 95% confidence intervals. It is evident that bees 1–18 (with five exceptions) are significantly right-biased, bees 80–102 (with two exceptions) are significantly left-biased, and bees 29–79 (with two exceptions) have no significant bias. These categories comprise 26.5% (right), 24.5% (left) and 49.0% (no bias) of the population of 102 tested bees. Thus, according to this method of bias analysis, approximately half of the bees are significantly biased, and this sample contains roughly equal numbers of left- and right-biased bees. The results for the Exit condition are shown in Fig 6B.

**Fig 7:**
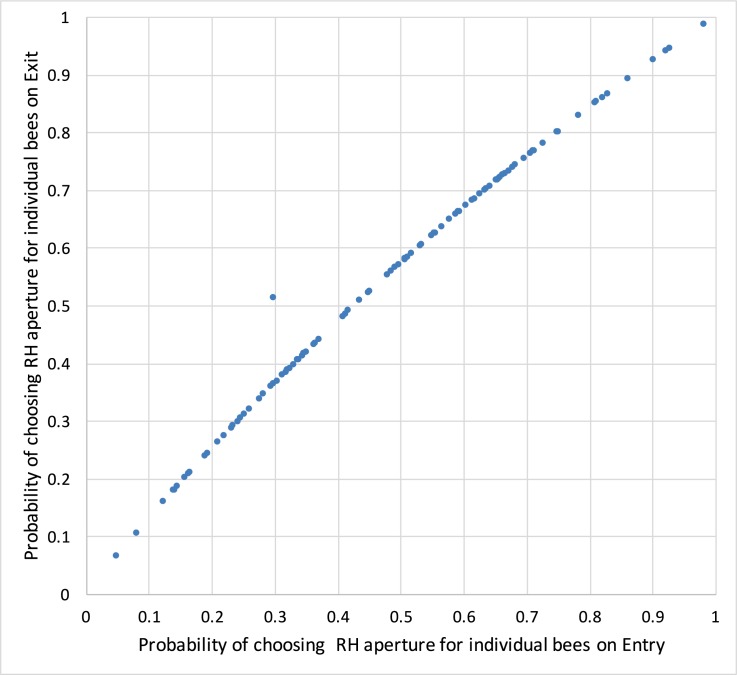
Equal apertures: Comparison of choice probabilities. Comparison of choice probabilities of individual bees for the right-hand (RH) aperture when both apertures are equally wide, for the Entry (horizontal axis) and Exit (vertical axis) conditions.

A comparison of Fig 6A and 6B reveals that the biases of individual bees are very consistent across the Entry and Exit conditions. This is examined more directly in Fig 7, which compares the choice probabilities of the individual bees for the right-hand aperture, in the Entry and Exit conditions. The values for individual bees are surprisingly consistent between Entry and Exit. Only one point (one bee) falls markedly outside the general trend, which is approximately a 45 deg line. The slightly upward-convex profile of the curve is consistent with the finding that the population bias during Exit is slightly (though not significantly) right-biased compared to that during Entry (see Fig 3 and Table 1).

Further inspection of Fig 7 also reveals that the rankings of the probabilities displayed by individual bees for choosing the right-hand aperture, in the Entry and Exit conditions, are remarkably consistent. Thus, the bee that displayed the strongest preference for the right-hand aperture on Entry is also the one that displayed the strongest preference for the right-hand aperture on Exit; this is also true for the bees that displayed the second strongest preference, third strongest preference, and so on down the line–except for one individual, corresponding to the outlier in the plot. The consistency in the rankings across the Entry and Exit conditions further supports the notion that, although the bias varies from one individual to the next, each individual displays a consistent bias across the Entry and Exit conditions.

It is of interest to inquire whether the shift from a marginal left bias during Entry to a marginal right bias during Exit for the overall population of bees is caused by the 9 individuals that exhibited a reversal of bias between the Entry and Exit conditions, as mentioned earlier. To examine this, we evaluated the mean bias for the Entry and Exit conditions when these 9 individuals are excluded from the analysis. We find that the mean bias (averaged over the remaining 93 bees) is 0.12 cm for the Entry condition (implying a marginal left bias) and -0.46 cm for the Exit condition (implying a marginal right bias). A similar analysis, conducted for the entire population of 102 bees, yields a mean bias of 0.13 cm for the Entry condition (again implying a marginal left bias) and -0.44 cm for the Exit condition (again implying a marginal right bias). There is no significant difference between the mean Entry biases or the mean Exit biases across the two groups of bees (P > 0.97). Thus, the transition from a weak left bias during Entry to a weak right bias during Exit is not caused by the 9 ‘reversers’; rather, it reflects the overall behavior of the entire population of 102 bees. The underlying reason for the overall shift in bias between Entry and Exit is that, for all of the 102 individuals tested, the probability of choosing the right-hand aperture when the two apertures are of equal width is slightly greater during Exit than during Entry, as shown in Fig 7.

Summarizing the findings of this section, we conclude that the reason for the near-zero bias in the overall population, as well as in the Entry and Exit conditions, is not that all of the bees are unbiased. Rather, roughly 50% of the bees are significantly biased, of which about half possess a right bias and the other half a left bias, resulting in an overall bias that is close to zero. Furthermore, individual bees display idiosyncratic levels of bias that are consistent in direction, irrespective of whether they are entering or leaving the apparatus.

### Quantitative modelling of discrimination capacity

We applied logistic regression models, with polychotomous independent variables, to obtain a better and more quantitative understanding of the factors that governed the bees’ choices between the two apertures. We examined the validity of four different models with increasing complexity. Model 1 hypothesizes that the bees’ choices depend only upon the relative widths of the two apertures; Model 2 proposes that the bees’ choices depend on aperture width as well as the identity of the bee (i.e. different bees possess different biases); Model 3 hypothesizes that the bees’ choices depend additionally on whether the bee is entering the tunnel or exiting it; and Model 4 postulates that, in addition to all of the factors considered in Model 3, the sensitivity to changes of aperture width depends upon whether the bee is entering or exiting the apparatus, in other words, there is an interaction between the sensitivity to aperture width and the condition of Entry/Exit. Details of the models are given in S1 Appendix.

Model 1 provides strong evidence that the aperture preference, averaged over all bees, is indeed affected by the relative aperture widths (P < 2*10–16).
Model 2 shows that the variation of aperture width is the primary determinant of aperture choice (P < 2*10–16). However, it also reveals that the bias parameters of 14 of the 102 individuals are significantly different from that of the grand mean of the entire population (P varying from 0.01 to 0.05), while the remaining individuals show no significant bias (P > 0.05). This analysis reinforces our conclusion about the presence of individuals with significant left or right biases, although the numbers are lower because the model uses a different approach from those used to derive the results shown in Figs 5 and 6.
Model 3 reveals that, in addition to the influences of varying aperture widths and individual varying biases, there is evidence that the bees’ aperture preference is also significantly affected by the Entry/Exit condition (P = 0.0009). Thus, bees make slightly different left versus right choices, depending upon whether they are entering or exiting a given aperture configuration, and this is caused by variation in bias strength. The negative value of Beta Entry (-0.29468; see Table A, S1 Appendix) indicates that, on average, the bees are slightly more right-biased in the Exit condition, as compared with the Entry condition. This is consistent with the results shown in Figs 3 and 7 and Table 1. Although the average bias displayed by bees is significantly different between the Entry and the Exit conditions, both of these values are small, and are close to zero.
Model 4 reveals that the sensitivity to aperture width does not depend upon whether the bees are entering or exiting the apparatus (P = 0.79).

Application of each of these models, in turn, to the data from the 102 bees reveals that, of Models 1–3, Model 3 is the best descriptor of the data, producing the smallest residuals (details in Table B, S1 Appendix). Model 4 does not provide a significant improvement over Model 3. Models 3 and 4 correctly predict the bees’ choices in 3491 (or 82%) of the total of 4253 choices that were recorded in this study (details in Table B, S1 Appendix).

The results of these analyses are in agreement with our conclusions that the factors that influence the bees’ choices are, primarily, (i) the relative widths of the apertures, (ii) the identity of the bee (and therefore its bias), and marginally, (iii) whether the bee is entering or exiting the apparatus.

### Transit times

**Fig 8:**
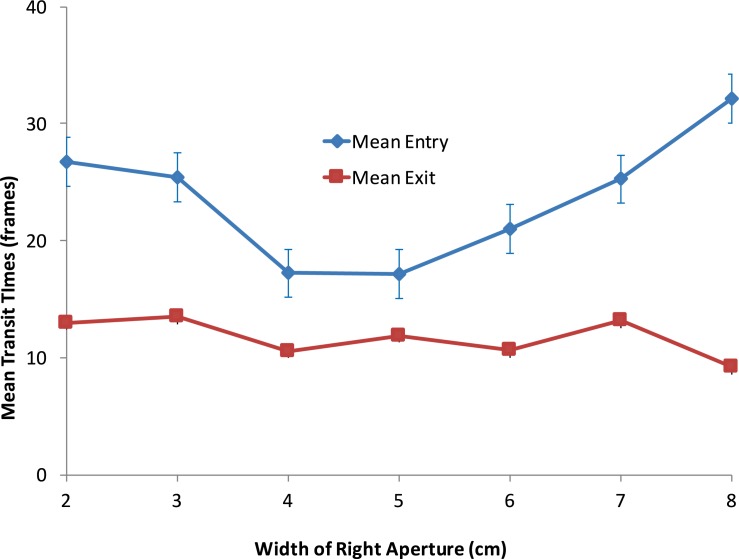
Variation of mean Entry and Exit transit times with the width of the right-hand aperture. n = 45 for both Entry and Exit data. Error bars denote standard error. The error bars for the mean Exit times are too small to be visible.

Does the average time required by a bee to choose one of the apertures and fly through it depend upon the aperture configuration? We measured the transit times of individual bees in the Entry condition as well as the Exit condition for each of the aperture configurations, as described in ‘Materials and methods’. The variation of the mean transit time with the width of the right-hand aperture is shown in Fig 8. For the Entry condition (blue curve) the transit time exhibits a U-shaped relationship, with the shortest transit times (17 frames) occurring when the two apertures are approximately equally wide. For the Exit condition (red curve), the mean transit times are much lower and are more or less constant, irrespective of the relative aperture widths.

**Fig 9:**
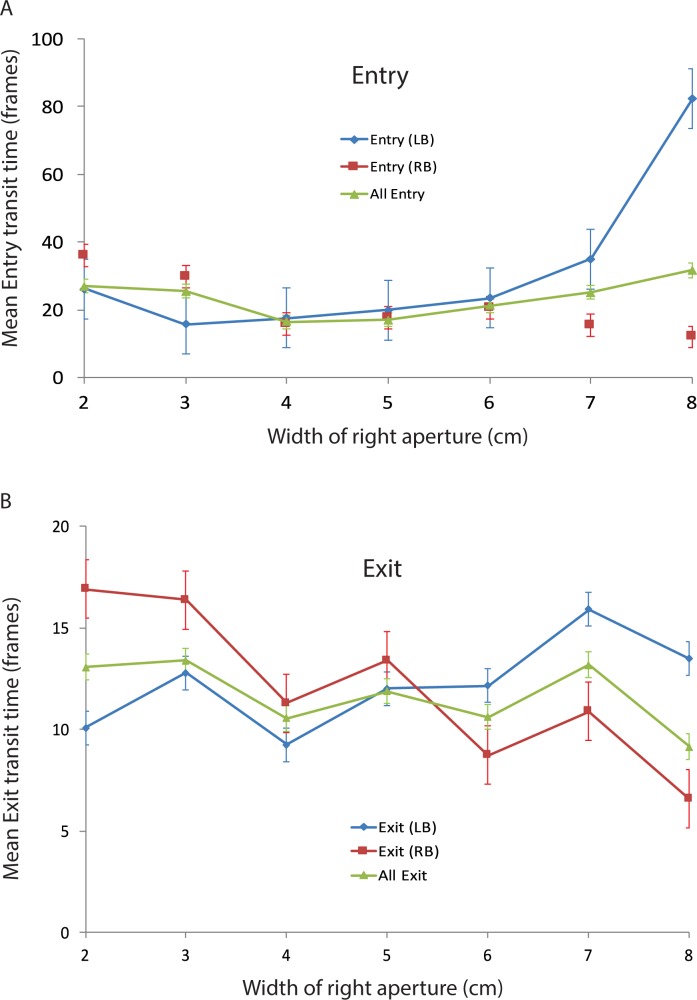
**A.** Variation of mean Entry transit times with the width of the right-hand aperture for left- and right-biased bees. Data are shown for left-biased bees (LB, n = 25), right-biased bees (RB, n = 20) and all bees (n = 45). Error bars denote standard error. **B.** Variation of mean Exit transit times with the width of the right-hand aperture for left- and right-biased bees. Data are shown for left-biased bees (LB, n = 17), right-biased bees (RB, n = 28) and all bees (n = 45). Error bars denote standard error.

Fig 9 shows the mean transit times recorded separately for the left- and right-biased bees, during Entry. In this simplified analysis, each bee was classified as being left-biased or right-biased according to whether its bias parameter ($-\frac{\beta_{0}}{\beta_{A}}$) was greater or smaller than 5.0 (see ‘Materials and methods‘), even though a number of bees in this set displayed biases that were close to zero. As the right aperture becomes progressively wider than the left aperture, the Entry times of the left-biased bees increase (Fig 9A), while those of the right-biased bees decrease. The overall transit time for the pooled data (left- and right-biased bees combined) is more or less independent of the relative aperture widths, because the transit times for the two groups of bees vary in a symmetrical and reciprocal fashion.

Fig 9B shows the corresponding data for the mean transit times of biased bees during Exit. These data display a similar pattern to that in Fig 9A, although the variations of transit time are less pronounced than in the Entry condition. Again, the transit time for the pooled data is more or less independent of the relative aperture widths, because the transit times for the left-biased and right-biased bees vary in a symmetrical and reciprocal fashion.

Overall, the transit times for the Exit flights are lower than for the Entry flights for each aperture configuration (P < 0.01, two-sample t-test), implying that the bees are flying faster when leaving the food source, and are also less hindered by narrow apertures.

To interpret the results shown in Figs 8 and 9, let us begin by considering what we might expect if all of the bees in the population were unbiased, so that the population, as a whole, is also unbiased. In this case, if the apertures are equally wide, roughly half of the bees will choose the left-hand aperture and the other half the right-hand aperture. Let us denote the average transit time under this condition by T*equal aperture*. If one aperture is wider than the other, then the bees would be more likely to choose to fly through the wider aperture, and so we may expect the average transit time in this condition, T*unequal aperture*, to be shorter than T*equal aperture*. Fig 8 shows that, for the Entry condition (blue curve), the transit time exhibits a U-shaped relationship, with the shortest transit times (17 frames) occurring when the two apertures are approximately equally wide. For the Exit condition (red curve), the mean transit times are much lower and are more or less constant, irrespective of the relative aperture widths. Both of these observations are contrary to the above expectation. This argues against the possibility that all of the bees in the population are unbiased.

Let us now consider a situation in which the overall population bias continues to be zero, but the population contains a significant proportion of bees that are individually biased to the left or to the right, as observed in Figs 5 and 6. If we were to test the right-biased bees with a range of aperture configurations, we would expect them to show a stronger preference for the right-hand aperture, than would the unbiased bees. This would cause the mean transit time of the right-biased bees to decrease as the right aperture is made wider, and increase as it is made narrower. Conversely, we would expect the mean transit time of the left-biased bees to increase as the right aperture is made wider, and decrease as it is made narrower. This is precisely what is observed in Fig 9.

Thus, the transit time data confirm our evidence for the existence of bees with individual biases, of either polarity, in the population of bees that was tested in this study: The transit time is longer if a bee’s intrinsic bias forces it to engage with the narrower aperture.

An interesting and related question concerns the behavior of unbiased versus strongly biased bees that confront two apertures of equal width. Do the unbiased bees require a longer time to make a choice between the two apertures? To examine this, we divided the bees whose transit times were measured, into two groups: (i) unbiased or weakly biased bees (bias magnitude ≤ 1.5 cm) and (ii) strongly biased bees (bias magnitude > 1.5 cm), and evaluated the mean transit time for each group when the apertures were of equal width. This calculation was done for the Entry condition as well as the Exit condition. In either condition, there was no significant difference between the mean transit times for the two groups, as shown Table 3. Therefore, unbiased or weakly biased bees require approximately the same amount of time to make a decision between two equally wide apertures, as do the strongly biased bees. Thus—interestingly, and perhaps contrary to what one might expect—weakly biased bees do not vacillate in front of the two equal apertures in a state of indecision.

**Table 3:** Comparison of average transit times of weak- and strongly-biased bees in the equal aperture condition.

Condition	Bias	Average transit time (frames)	t-test for significant difference
Entry (44 bees)	Weak (23 bees)	17.9	P = 0.74 (n.s.)
	Strong (21 bees)	18.8	
Exit (40 bees)	Weak (20 bees)	14.3	P = 0.14 (n.s.)
	Strong (20 bees)	10.5	

## Discussion

The challenge of finding a suitable route through a cluttered environment is faced not just by honeybees, but by a number of animal species, including birds, that inhabit dense vegetation [14, 20]. Toads, for example, are able to gauge the width of a gap between two obstacles from afar, and decide whether to go through the gap or circumvent it, depending upon its width [21].

Our study reveals that honeybees, when confronted with two vertically oriented apertures, show a tendency to choose to fly through the wider aperture. It is known that flight through narrow passages is more time-consuming [3], hence choosing the wider gap should increase foraging efficiency. Moreover, the risk of wing damaging collisions that can impose significant costs on the bees’ flight performance and survival [5, 22] should be lower in an opening that is wider and easier to negotiate.

The bees in our experiment are clearly sensitive to differences in aperture width: The wider aperture is chosen about 80% of the time, on average, if it is twice as wide as the narrower aperture. Two kinds of cues that the bees could have been using to discriminate aperture widths are (i) the optic flow generated by the edges of the aperture as it is approached and (ii) the relative brightness of the two apertures: Phototaxis could cause the bees to move toward the wider aperture, as has been shown for orchid bees [2]. While our experiment does not allow us to distinguish between these two possibilities and was not designed with the intention to do so, it is unlikely that bees used phototactic cues to discriminate between the two openings during Entry flights. Firstly, the illumination inside the tunnel was uniform across the left and right-hand sides of the tunnel, and, secondly, bees that were flying towards the feeder viewed both apertures against a textured background wall inside the setup, and not against the open sky. However, phototaxis could have played a role in the Exit flights, as detecting and discriminating the two apertures could be easier when they are positioned in front of the bright tunnel opening. Along with a high motivation to return to the hive as quickly as possible after foraging, this could explain why the transit times for the Exit flights were much shorter and less variable than the Entry flights. Hoenicke et al. [23] found a similar pattern in the ant *Formica pratensis*: Ants returned to the nest with a higher velocity compared to foragers leaving the nest, ensuring that food reaches the colony quickly and efficiently.

While bees generally prefer the wider aperture, our principal and unexpected finding is that they display individually varying, idiosyncratic biases. It is important to note in this context, that a bee’s choice of the left or the right aperture was not simply a consequence of her position in the tunnel prior to encountering the barrier: Bees tend to fly along the midline of a corridor, equidistant to both side walls, balancing optic flow (‘centering response’, [24]). This is particularly true for an experimental design like ours, with a relatively narrow tunnel and entrance and feeder positioned along the midline [25], and identical patterns on the two side walls.

In our study, the biases of individual bees vary in strength and direction, ranging from an extreme left preference, through zero bias, to an extreme right preference. Apart from a few individuals (n = 9), each bee displays its individual bias consistently, both while entering the apparatus and while leaving it. This is different to the conditional lateralization of *F*. *pratensis*, in which inbound individuals prefer to walk on the left side of their foraging trail, while outbound ants do not display individual lateralization [23].

Our tests reveal a substantial and equal number of left-biased and right-biased bees (about 22.5% in each case, with the exact value depending upon the method of analysis) among the 102 individuals that were tested. The population as a whole displays no significant bias, because the polarities and the strengths of the biases of the individual bees are close to mirror-symmetrical (Figs 4–6), and therefore cancel each other out. This is the first discovery of individually varying lateralization of behavior in honeybees.

Most of the well-studied and well-known instances of behavioral lateralization in animals involve biases that exist at the level of the population. That is, all (or most) of the individuals exhibit a bias in the same direction. Among the vertebrates, some examples include right-handedness and right-eye dominance in humans [26], the use of the right eye for detecting food and the left eye for detecting predators in chickens [27] and toads [28, 29], use of the right forelimb to initiate climbing in green tree frogs [30] and detour behavior in fish [31].

It has been suggested that lateralization at the population level could have evolved to benefit social species, i.e. animals that live and interact within groups [9]. For example, it has been hypothesized that schooling fish reduce individual risk to predation by ensuring that most individuals turn in the same direction in response to the appearance of a predator. By the same token, individuals of a non-schooling species of fish could benefit by having individually varying biases (some left, others right), because a predator could then never predict the direction in which its prey would turn [9]. Some support for this hypothesis is provided by Bisazza et al. [32]. Their analysis of the escape behavior of 16 fish species revealed that gregarious species showed a consistent population bias in turning preferentially left or right in response to a dummy predator, while non-gregarious species were lateralized at the individual level.

More recently there has been growing evidence to indicate that, at least in the context of some behaviors, biases can vary across individuals within a population: some individuals can be left-based, others right-biased, and yet others unbiased. Such a mix of lateralization is typical for many fish schools, however the extent to which individual laterality determines group organization and the position of fish within their school can differ between species and sexes [33]. Interspecific differences in lateralization have also been reported in cephalopods [34]. Other examples of lateralization at the individual or species level include handedness in chimpanzees [35], tool use in crows [36, 37], and species-dependent variation in the foot that is used for picking up food in certain parrot species [38].

Furthermore, biases can change depending upon the task that an animal is performing. For example, mice [39] and marmosets [40] display a change in bias at the population level when a simple reaching task is modified slightly; individual budgerigars exhibit changes in their choice of landing site, depending upon whether they encounter a single long perch, or two short perches positioned side by side [13]. The significance or evolutionary origin of individually varying bias is as yet unknown in most cases, and is largely a subject of conjecture. But some individual differences in behavior can be explained by anatomy–for example, asymmetrical body structure (e.g. fiddler crabs: [41]; crayfish: [42]). In schools of fish, a high variability of laterality in phenotypes presumably entails fitness benefits, as the positioning of individual fish within the group optimizes the detection of predators and prey and increases school coordination and cohesion [33].

Among the invertebrates, the majority of biases that have been recorded so far are manifest at the population level, regardless of whether the species is social or solitary [18]. However, the few documented instances of individual lateralization have all been found primarily in solitary species ([18], Table 1). One clear example of individual lateralization is the turning behavior of the seven-spotted ladybird, *Coccinella septempunctata*, where individuals display distinct left, zero or right biases in their choice of branches while foraging for aphids on plants [11]. Interestingly, about 45% of the individuals showed a turning bias, with similar numbers biased to the left or the right, as in our honeybee study. That ladybird study also demonstrates, through simulations, that biased individuals would forage more efficiently than unbiased ones. While the modelling does not elucidate the potential benefits of having individuals with different biases in the population, one advantage might be that it promotes a symmetrical and therefore more complete coverage of the plant, with less competition for food sources on the same side. Drosophila [12] display individually lateralized choice preferences in Y mazes, although the functional significance of this finding remains unclear. Walking locusts exhibit individual preferences with regard to which foreleg is first used to reach across a gap to be traversed [43]. Strongly biased individuals performed better when crossing the gap and made fewer reaching errors due to improved motor control [44]. Adult crayfish display individually lateralized escape responses, with the direction of the bias depending on the morphology of the carapace [41]. This unpredictability may be advantageous in thwarting attacks from predators.

In honeybees, all of the biases that have been recorded so far occur at the population level. Examples include better learning of visual stimuli through the right-hand visual pathway [45], preferential use of the right antenna for contact with conspecifics [46], and differences in the learning of olfactory stimuli between the left and the right olfactory pathways [19, 47, 48]. Ours is the first discovery of individually varying behavioral lateralization in honeybees, and is contrary to the expectation of a population-level bias in this highly social creature.

What could be the functional significance of the individually varying bias that bees display when faced with a choice of apertures? One possibility is that this pattern of biases enhances the speed and safety with which a group of honeybees can fly through dense foliage–a situation that can be encountered when a group of bees forages at a food site, or a swarm moves to a new home. Let us consider a (simplified) scenario in which a group of bees is faced with a choice of flying through one of two clear passages through a thicket of branches. It would be detrimental if all the bees possessed the same bias, say, toward the left, as a population bias of this kind would make all individuals try to fly through the left-hand passage, thus blocking each other, and slowing down as well as endangering the passage of the swarm through the thicket. In this case, the right-hand passage would not be used at all, and therefore would be wasted. On the other hand, it would also not be beneficial to have zero bias in each of the bees, because if the two passages were of unequal size, a group of unbiased bees would all try to fly through the wider passage, overcrowding it and again slowing down progress. The narrower passage would not attract any bees even if it were wide enough to permit safe flight, and it would thus be a ‘waste’ of a potentially useful conduit. On the other hand, if, say, half the population was left-biased and the other half right-biased, two passages of equal width would attract roughly equal numbers of bees, thus speeding up the progress of the swarm through the thicket. Furthermore, the left-biased and right-biased bees would choose their preferred passage without any hesitation, leading to a quicker and safer passage of the flock. In this case, as the right-hand passage is gradually made wider than the left-hand passage, all the bees would not immediately flock to the right-hand passage: Many of the left-biased bees would continue to favor the left-hand passage until it became too narrow for safe transit. Thus, a hybrid flock of left and right-biased bees would make better use of both of the available routes, and fly through the thicket more quickly. The same benefits could apply at a busy hive entrance, a more complex situation with a large number of individuals flying in opposite directions (in and out) at the same time in a relatively confined area. It can be shown that the same considerations also apply to situations that involve choosing among multiple apertures [14]. Our hypothesis assumes that bees in a group act independently of each other when making their aperture choices–an assumption that we have not tested in the present study, as we had to record the bees’ behavior when they made their choices in isolation in order to identify their inherent bias.

Bhagavatula et al. [14] present a mathematical model that captures these considerations quantitatively, and demonstrates that a group of individuals will pass through a double-aperture configuration most efficiently (in the minimum time) if the individuals in the group carry a range of different biases–distributed uniformly ranging from an extreme left-bias, through no bias, to an extreme right-bias. With a uniform distribution of biases, the number of individuals that choose each aperture would be proportional to the width of that aperture. Consequently, both apertures would be used effectively, and would finish clearing the bees at the same time. This uniform distribution of biases is close to what the bees exhibit in our experiments. The model in [13] was developed in the context of investigating how birds (budgerigars) choose between two apertures, in a study analogous to the present one. That study found that budgerigars display individually varying left-right biases, as do honeybees, but it was restricted to a sample of 7 birds. Our present study, involving 102 individually marked honeybees, provides a stronger validation of the model.

Our study does not investigate the effect of learning and previous experience on the bees’ route choices, which would be an interesting topic for future investigation. Kells & Goulson [49] found that foraging bumblebees display handedness and tend to rotate in the same direction on successive inflorescences. One explanation could be that the bumblebees simply repeat a behavior that has proved to be successful. For the honeybees in our experimental paradigm, however, it would not necessarily be beneficial to stick to the side that was previously successful. This is because we changed the aperture configuration randomly every five minutes, which means that a bee’s choice on its first flight (e.g. left aperture, because it is wider) may not necessarily be the appropriate choice on her next visit (for example, if the wider aperture is now on the right). The chances of a given bee revisiting the same aperture configuration (before it is changed) are therefore very small.

In a honeybee colony, the queen typically mates with several drones. Consequently, worker (female) bees could have different fathers (drones), and the individual lateralization that we have observed could arise from idiosyncratic, genetically-based asymmetries in the sensorimotor pathways, derived from different fathers. Whether this is actually the case, and if so, whether these asymmetries are reinforced through repeated execution of the behaviors, would be interesting topics for future research.

In summary, while honeybees and budgerigars are social species, our studies with these species are revealing that they can and do exhibit individually varying lateralization, depending upon the behavior being executed and the context in which the behavior occurs.

## Supporting information

S1 Appendix This is the entire document containing the suppprting information. (DOCX)
