# Supplementary material for: Obstacle traversal and route choice in flying honeybees: Evidence for individual handedness
Source: PLoS One. 2017 Nov 2;12(11):e0184343. doi: 10.1371/journal.pone.0184343 (PMC5667806; doi:10.1371/journal.pone.0184343)
Supplement: S1 Appendix — (DOCX) [file pone.0184343.s001.docx]

**S1 APPENDIX**

**Obstacle traversal and route choice in flying honeybees:**

**Evidence for individual handedness**

**Marielle Ong, Michael Bulmer, Julia Groening, and Mandyam V. Srinivasan**

**Models characterizing aperture choice**

As mentioned in ‘Results’, four different logistic regression models, with polychotomous independent variables, were postulated to account for the factors that influence the bees’ choices. The model parameters were fitted to the data using an R program in the statistics toolbox that implements maximum-likelihood parameter estimation.

1. *Model 1*

Model 1 hypothesizes that the bees’ choices depend only upon the relative widths of the two apertures. This model is characterized by the equation

$y=ln\left( \frac{p}{1-p} \right)=\beta_{0}+\beta_{A}x_{A}$ (1)

where p is the probability of a bee choosing the right hand aperture given a particular right-hand aperture width $x_{A}$, which varies from 2 cm to 8 cm. The dependent variable, y, is expressed as the log odds ratio of the probability of a right hand choice to a left hand choice (1).

1. Can be re-expressed as

$p=\frac{1}{\left[ 1+e^{{-\beta}_{A}\left( x+\frac{\beta_{0}}{\beta_{A}} \right)} \right]}$ (2)

which is the logistic form given in ‘Methods’, and graphed in Fig. 14.

$\beta_{A}$ defines the slope of the function, which is a measure of the sensitivity of the bees’ choices to changes in the width $x_{A}$ of the right-hand aperture. The ratio $\frac{\beta_{0}}{\beta_{A}}$ defines the position of the sigmoidal function along the horizontal axis. $\beta_{0}$ and $\beta_{A}$ are fitted to the data using the R program mentioned above. This model is fitted to the entire data set (Entry and Exit flights), without discriminating between the two conditions.

1. *Model 2*

Model 2 extends the general logistic regression to include the effect of the bias of each individual bee on the mean choice probability. This can be expressed as

 (3)

Where $\beta_{0}$ and $\beta_{A}$ are as in (2), and the parameters $\beta_{c}$ specify the difference in the bias parameter of each individual bee c from the population bias parameter $\beta_{0}$. $x_{c}$ is a binary indicator variable ($x_{c}$ = 1 or 0) that specifies the inclusion or exclusion of individual bees in the regression, where $c$ represents the bee’s color identification code. Setting $x_{c}$ = 1 includes bee $c$ in the regression, and setting $x_{c}$ = 0 excludes it. Each bee $c$ is associated with a bias correction parameter $\beta_{c}$, which quantifies the bias of the individual by specifying its departure from the global bias parameter $\beta_{0}$ (grand-mean coding is used). By setting $x_{c}$ = 1 for a particular bee c and $x_{d}$ = 0 for the remaining bees $($d ≠ c$)$, the regression procedure can be used to estimate the bias of each individual bee $c$, in turn. The value of $\beta_{A}$ is assumed to be the same for all bees. In effect, we assume that different bees transition from the left-hand aperture to the right-hand aperture at different right-hand aperture widths, but that the rate of transition is the same for each bee at the point of transition, irrespective of whether it is entering or exiting the apparatus.

1. *Model 3*

Model 3 elaborates on Model 2 by allowing for differences in choice behavior between the Entry and Exit conditions. This is accomplished by adding another term, $\beta_{\mathrm{Entry}}x_{\mathrm{Entry}}$, resulting the logistic regression model

 (4)

Here $x_{\mathrm{Entry}}$ is a binary indicator variable that specifies the condition of Entry ($x_{\mathrm{Entry}}$ = 1) or Exit ($x_{\mathrm{Entry}}$ = 0). For the Exit condition, $\beta_{0}$, $\beta_{A}$ and the $\beta_{c}$ are computed as in (3). For the Entry condition $\beta_{\mathrm{Entry}}$ specifies the correction that should be applied to the computed Exit bias parameter $\beta_{0}$.

As in Model 2, this model assumes that the sensitivity to aperture widths $\beta_{A}$ is the same for all individuals, regardless of their bias, and regardless of whether they are entering or exiting the apparatus.

1. *Model 4*

Model 4 elaborates on Model 3 by allowing for the possibility that the sensitivity $\beta_{A}$, to the variation of aperture widths differs depending on whether a bee is entering or exiting the apparatus. This is accommodated by adding a term $\beta_{Entry-Aperture}{.x}_{Entry.}x_{A}$ , where $x_{\mathrm{Entry}}$is the binary Entry-Exit indicator variable as used in Model 3:

(5)

For the Exit condition, $x_{\mathrm{Entry}}$ = 0, and $\beta_{0}$, $\beta_{A}$ and the $\beta_{c}$ are computed as in (3). For the Entry condition, $\beta_{\mathrm{Entry}}$ specifies the correction that should be applied to the computed Exit bias parameter $\beta_{0}$, and $\beta_{Entry-Aperture}$ specifies the correction that should be applied to the computed Exit sensitivity parameter $\beta_{A}$.

**Validation of different mathematical models**

The validity of each model was measured using classification tables, which display the number and percentage of the observed choices that were predicted correctly by the model. The results are shown in Table A. For instance, out of 2103 left choices observed, Model 1 correctly predicted 1503, or 71.47% of the left choices. Model 2 performs better than Model 1, correctly predicting 81.12% of the left choices. Model 3 performs slightly better than Model 2, correctly predicting 81.46% of the left choices. Model 4 shows the same performance as Model 3, consistent with our statement above that there is no interaction between aperture width and Entry/Exit condition.

Models 3 and 4 display very similar results, which are therefore summarized together. This supports the notion that the addition of the interaction variable between aperture width and Entry/Exit conditions does not increase the number of correctly predicted choices.

**Table A.** Bias parameters for Model 3. The table shows values for the Intercept (β0), Aperture (βA), Entry (β*Entry*) and individual bees (Bee*ijk*).

Significance: ‘***’: p < 0.001; ‘**’: p < 0.01; ‘*’: p < 0.05, ‘.’: p < 0.1, ‘ ’: p > 0.1

| Coefficients | Estimate | Std. | Error | Pr(>\|z\|) | Significance |
| --- | --- | --- | --- | --- | --- |
| $\boldsymbol{\beta}_{\boldsymbol{0}}$ | -5.2338 | 0.56171 | -9.318 | <2E-16 | *** |
| $\boldsymbol{\beta}_{\boldsymbol{A}}$ | 1.02732 | 0.03045 | 33.735 | <2E-16 | *** |
| $\boldsymbol{\beta}_{\boldsymbol{Entry}}$ | -0.29468 | 0.08881 | -3.318 | 0.000906 | *** |
| BeeBBj | 0.19557 | 0.62959 | 0.311 | 0.756083 |  |
| BeeBG | -0.81173 | 0.62883 | -1.291 | 0.196755 |  |
| BeeBgi | -1.56813 | 0.74095 | -2.116 | 0.034314 | * |
| BeeBN | 1.02263 | 0.68568 | 1.491 | 0.135856 |  |
| BeeBO | 0.42314 | 0.71288 | 0.594 | 0.552798 |  |
| BeeBpi | -0.93647 | 0.84805 | -1.104 | 0.269479 |  |
| BeeBPk | 1.67575 | 0.62654 | 2.675 | 0.007481 | ** |
| BeeBRb | 0.74959 | 0.77693 | 0.965 | 0.334636 |  |
| BeeBWb | 1.85183 | 1.01473 | 1.825 | 0.068008 | . |
| BeeByj | 1.08622 | 0.83594 | 1.299 | 0.193803 |  |
| BeeGBk | 0.94843 | 0.6736 | 1.408 | 0.15913 |  |
| BeeGGk | -0.28173 | 0.63852 | -0.441 | 0.659051 |  |
| BeeGlG | -0.75571 | 0.8276 | -0.913 | 0.36117 |  |
| BeeGN | 0.65493 | 0.79951 | 0.819 | 0.412697 |  |
| BeeGNb | 0.85743 | 0.82602 | 1.038 | 0.299259 |  |
| BeeGoi | 0.70908 | 0.77318 | 0.917 | 0.35909 |  |
| BeeGok | 0.15244 | 0.67829 | 0.225 | 0.822183 |  |
| BeeGPk | -0.46396 | 0.60379 | -0.768 | 0.44224 |  |
| BeeGR | 0.51426 | 0.67997 | 0.756 | 0.449476 |  |
| BeeGW | 0.76646 | 0.81905 | 0.936 | 0.349377 |  |
| BeeGwi | 1.10945 | 1.00822 | 1.1 | 0.271158 |  |
| BeeNB | 1.48903 | 0.71988 | 2.068 | 0.038598 | * |
| BeeNO | -0.16423 | 0.72772 | -0.226 | 0.821457 |  |
| BeeNod | 1.2974 | 0.82007 | 1.582 | 0.113636 |  |
| BeeNPb | -0.87469 | 0.71611 | -1.221 | 0.221917 |  |
| BeeNPc | -0.72395 | 0.74658 | -0.97 | 0.332206 |  |
| BeeNPh | 1.03976 | 0.72189 | 1.44 | 0.149774 |  |
| BeeNRb | 1.1593 | 0.68856 | 1.684 | 0.092245 | . |
| BeeNV | 0.90884 | 0.68243 | 1.332 | 0.182935 |  |
| BeeNVc | -0.28859 | 0.84486 | -0.342 | 0.732663 |  |
| BeeNwa | 0.36015 | 0.65285 | 0.552 | 0.581176 |  |
| BeeNWb | 1.83979 | 0.71606 | 2.569 | 0.01019 | * |
| BeeNyh | 0.94069 | 0.67445 | 1.395 | 0.16309 |  |
| BeeOG | -1.28259 | 0.71683 | -1.789 | 0.073574 | . |
| BeeOgi | -1.42684 | 0.80467 | -1.773 | 0.076197 | . |
| BeeOni | -0.1743 | 0.822 | -0.212 | 0.832075 |  |
| BeeOrd | -0.25371 | 0.9047 | -0.28 | 0.77914 |  |
| BeeOW | -0.25517 | 0.73223 | -0.348 | 0.727478 |  |
| BeeOwi | -1.41649 | 0.73216 | -1.935 | 0.053032 | . |
| BeePB | -0.57592 | 0.87345 | -0.659 | 0.509662 |  |
| BeePBj | 0.13066 | 0.76285 | 0.171 | 0.864 |  |
| BeePBk | -0.2387 | 0.7422 | -0.322 | 0.747747 |  |
| BeePGb | -1.22274 | 0.74539 | -1.64 | 0.100921 |  |
| BeePGk | 2.95717 | 0.65477 | 4.516 | 6.29E-06 | *** |
| BeePP | 0.3088 | 0.66131 | 0.467 | 0.640534 |  |
| BeePPh | 1.9648 | 0.90929 | 2.161 | 0.03071 | * |
| BeePPj | 0.37884 | 0.86865 | 0.436 | 0.662745 |  |
| BeePPk | 1.04627 | 0.59787 | 1.75 | 0.080118 | . |
| BeePR | -1.04116 | 0.76668 | -1.358 | 0.174457 |  |
| BeePSk | -0.70058 | 0.63457 | -1.104 | 0.269584 |  |
| BeePWj | 0.30721 | 0.60745 | 0.506 | 0.613044 |  |
| BeePWk | 1.36841 | 0.78321 | 1.747 | 0.080606 | . |
| BeePYb | 0.18685 | 0.77478 | 0.241 | 0.809426 |  |
| BeePyj | -1.23745 | 0.67032 | -1.846 | 0.064886 | . |
| BeeRGk | -0.39317 | 0.63197 | -0.622 | 0.533854 |  |
| BeeRN | 2.60859 | 0.97595 | 2.673 | 0.00752 | ** |
| BeeRni | 4.42777 | 0.95099 | 4.656 | 3.22E-06 | *** |
| BeeRO | -0.14148 | 0.85856 | -0.165 | 0.869115 |  |
| BeeRoc | -2.04692 | 1.15278 | -1.776 | 0.075793 | . |
| BeeRP | 0.77321 | 0.72788 | 1.062 | 0.288107 |  |
| BeeRPj | -0.65415 | 0.61799 | -1.059 | 0.289818 |  |
| BeeRRj | 1.06632 | 0.7087 | 1.505 | 0.132426 |  |
| BeeRVk | 1.13737 | 0.78721 | 1.445 | 0.148513 |  |
| BeeRW | 0.59112 | 0.79008 | 0.748 | 0.454347 |  |
| BeeRWk | -0.43803 | 0.89643 | -0.489 | 0.625096 |  |
| BeeRyj | 0.41881 | 1.20871 | 0.346 | 0.728973 |  |
| BeeRyk | 0.8756 | 0.62177 | 1.408 | 0.159063 |  |
| BeeSok | 1.91034 | 0.64236 | 2.974 | 0.00294 | ** |
| BeeSPk | -0.31733 | 0.65249 | -0.486 | 0.626726 |  |
| BeeSSj | -0.37403 | 0.67472 | -0.554 | 0.579333 |  |
| BeeSSk | 0.45931 | 0.65529 | 0.701 | 0.483349 |  |
| BeeSWk | 1.96721 | 0.7198 | 2.733 | 0.006276 | ** |
| BeeVBk | 0.97792 | 0.61447 | 1.591 | 0.111497 |  |
| BeeVP | -1.37263 | 0.74746 | -1.836 | 0.0663 | . |
| BeeVRk | 0.03591 | 0.74475 | 0.048 | 0.961539 |  |
| BeeVWk | 0.61195 | 0.76773 | 0.797 | 0.425403 |  |
| BeeWbi | 0.81723 | 0.76029 | 1.075 | 0.282423 |  |
| BeeWGj | 0.60632 | 0.69886 | 0.868 | 0.385623 |  |
| BeeWN | -0.48042 | 0.66233 | -0.725 | 0.468241 |  |
| BeeWO | 0.05855 | 0.69976 | 0.084 | 0.933317 |  |
| BeeWPk | 1.0292 | 0.60745 | 1.694 | 0.090209 | . |
| BeeWSk | -0.72558 | 0.66555 | -1.09 | 0.275627 |  |
| BeeWV | 2.85772 | 0.72777 | 3.927 | 8.61E-05 | *** |
| BeeWWj | -0.5392 | 0.57447 | -0.939 | 0.347928 |  |
| BeeWY | 0.43427 | 0.85975 | 0.505 | 0.613481 |  |
| BeeWYb | -0.36186 | 0.83193 | -0.435 | 0.663591 |  |
| BeeWyi | 0.02207 | 0.70442 | 0.031 | 0.975 |  |
| BeeYgk | -0.46189 | 0.75794 | -0.609 | 0.542261 |  |
| BeeYN | 0.52353 | 0.8217 | 0.637 | 0.524041 |  |
| BeeYnb | 0.3339 | 0.67301 | 0.496 | 0.619809 |  |
| BeeYni | 1.2734 | 0.81906 | 1.555 | 0.120016 |  |
| BeeYpi | -1.06037 | 0.80318 | -1.32 | 0.186767 |  |
| BeeYpk | -0.16598 | 0.59083 | -0.281 | 0.77877 |  |
| BeeYri | -0.34512 | 0.83714 | -0.412 | 0.680149 |  |
| BeeYrk | 1.48424 | 0.59933 | 2.476 | 0.013268 | * |
| BeeYWb | 2.22452 | 0.85336 | 2.607 | 0.00914 | ** |
| BeeYwc | 0.90889 | 0.86621 | 1.049 | 0.294053 |  |
| BeeYwi | -0.78971 | 0.79359 | -0.995 | 0.31968 |  |
| BeeYyc | -0.22584 | 0.81844 | -0.276 | 0.782596 |  |
| BeeYyh | 1.28645 | 0.88374 | 1.456 | 0.145478 |  |
| BeeYyj | 1.21918 | 0.63412 | 1.923 | 0.054525 | . |
| BeeYyk | -2.56055 | 0.72402 | -3.537 | 0.000405 | *** |

**Table B.** Performance of Models 1-4

| Aperture | Total number of observed choices | Number of choices correctly predicted by model | Percentage of choices correctly predicted by model |
| --- | --- | --- | --- |
| Model 1 | | | |
| left | 2103 | 1503 | 71.47% |
| right | 2150 | 1788 | 83.16% |
| Model 2 | | | |
| left | 2103 | 1706 | 81.12% |
| right | 2150 | 1780 | 82.79% |
| Models 3 and 4 | | | |
| left | 2103 | 1713 | 81.46% |
| right | 2150 | 1778 | 82.70% |
